# Charles Ferrier Walters

**Published:** 1945

**Authors:** 


### CHARLES FERRIER WALTERS, F.R.C.S.

ON 24th December, 1944, Mr. C. Ferrier Walters died in Bristol after an illness which had lasted several months. He was at the time of his death Honorary Consulting Surgeon to the Bristol Royal Hospital and Group Officer to the Bristol Area under the Ministry of Health E.M.S. Scheme. Mr. Walters was born on 26th December, 1874, the son of Thomas Walters, Chartered Accountant. He was educated at Burton College, where his uncle, Rev. Dr. Ferrier, was headmaster. On leaving school he was for a short time apprenticed to a dental surgeon in Park Street, Bristol. But he soon changed his mind and entered the Medical School of University College, Bristol. His Clinical work was done at the Bristol Royal Infirmary and he qualified M.R.C.S., L.R.C.P. in 1900, having passed the first F.R.C.S. examination. After holding resident posts at the Bristol Royal Infirmary, he passed the Final Examination and obtained the F.R.C.S. in 1904.

Mr. Walters was appointed Assistant Surgeon at the Bristol Royal Infirmary in 1909, and Surgeon in 1915, rapidly becoming one of our busiest surgeons. In 1929 he was President of our Society, choosing as his subject for the Presidential Address "The Duty of Medicine to the Race." He was President of the Bath, Bristol and Somerset Branch of the British Medical Association in 1932–33.

